# Accounting for seasonal patterns in syndromic surveillance data for outbreak detection

**DOI:** 10.1186/1472-6947-6-40

**Published:** 2006-12-04

**Authors:** Tom Burr, Todd Graves, Richard Klamann, Sarah Michalak, Richard Picard, Nicolas Hengartner

**Affiliations:** 1 Statistical Sciences, Mail Stop F600, Los Alamos National Laboratory, Los Alamos, NM 87545, USA; 2 Discrete Simulation Sciences, Mail Stop M997, Los Alamos National Laboratory, Los Alamos, NM 87545, USA

## Abstract

**Background:**

Syndromic surveillance (SS) can potentially contribute to outbreak detection capability by providing timely, novel data sources. One SS challenge is that some syndrome counts vary with season in a manner that is not identical from year to year.

Our goal is to evaluate the impact of inconsistent seasonal effects on performance assessments (false and true positive rates) in the context of detecting anomalous counts in data that exhibit seasonal variation.

**Methods:**

To evaluate the impact of inconsistent seasonal effects, we injected synthetic outbreaks into real data and into data simulated from each of two models fit to the same real data. Using real respiratory syndrome counts collected in an emergency department from 2/1/94–5/31/03, we varied the length of training data from one to eight years, applied a sequential test to the forecast errors arising from each of eight forecasting methods, and evaluated their detection probabilities (DP) on the basis of 1000 injected synthetic outbreaks. We did the same for each of two corresponding simulated data sets. The less realistic, nonhierarchical model's simulated data set assumed that "one season fits all," meaning that each year's seasonal peak has the same onset, duration, and magnitude. The more realistic simulated data set used a hierarchical model to capture violation of the "one season fits all" assumption.

**Results:**

This experiment demonstrated optimistic bias in DP estimates for some of the methods when data simulated from the nonhierarchical model was used for DP estimation, thus suggesting that at least for some real data sets and methods, it is not adequate to assume that "one season fits all."

**Conclusion:**

For the data we analyze, the "one season fits all " assumption is violated, and DP performance claims based on simulated data that assume "one season fits all," for the forecast methods considered, except for moving average methods, tend to be optimistic. Moving average methods based on relatively short amounts of training data are competitive on all three data sets, but are particularly competitive on the real data and on data from the hierarchical model, which are the two data sets that violate the "one season fits all" assumption.

## Background

This paper focuses on syndromic surveillance (SS), an example of which is the frequent (usually daily) monitoring of counts of patient visits categorized into syndromes. The categorization arises upon mapping patient chief complaint and/or diagnosis data to syndrome categories such as respiratory, neurological, or gastrointestinal. The definition of SS also includes non-clinical data sources such as pharmacy sales, absenteeism, nurse hotline calls, etc. Although we focus on syndrome counts, seasonal patterns are sometimes present in these other SS data sources.

For overviews of SS, see reports from the 2004 National SS Conference in the Morbidity and Mortality Weekly Report [1] or publications available from [2].

SS can potentially contribute to outbreak detection capability by providing novel, timely data sources. However, concerns remain regarding signal to noise ratios, costs, maintainability, etc. [3]. One SS challenge is that some syndrome counts (such as respiratory) vary with season in a manner that is not consistent from year to year. Our goal is to evaluate the impact of inconsistent seasonal effects on performance assessments (false and true positive rates) in the context of detecting anomalous counts in data that exhibit seasonal variation.

To study the impact of inconsistent seasonal effects, we injected synthetic outbreaks into real data and two types of simulated data with attention to performance evaluation issues that arise due to violation of the "one season fits all" assumption. The informal term "one season fits all" describes any model that assumes each year's seasonal peak has the same onset, duration, and magnitude. For the simulated data, we included (1) a nonhierarchical model that assumes "one season fits all," and (2) a more realistic hierarchical model that captures violation of this assumption. We then evaluated the performance of several forecasting methods, including a method not considered in earlier studies such as [4,5]. Each forecast method has corresponding forecast errors defined as the difference between the actual counts and the forecasted (predicted) counts. Forecast errors are monitored to detect the injected outbreaks. Performance is defined here as the outbreak detection probability (DP) for a small false positive rate of one false alarm per year.

The following sections describe the data, the hierarchical and nonhierarchical models, our study and its results, and finally, provide conclusions.

## Methods

### Real rata: Emergency Center daily respiratory counts

We use data from the Emergency Center of the University Hospital, Albuquerque NM (a tertiary-care county-university health sciences center) collected from the BSafer system [6]. The data is from the computerized patient tracking system in place since 1994. We consider only those daily counts for which the chief complaint was mapped into the respiratory category.

### Simulated data: nonhierarchical modeling with over-dispersion

Let *C*(*d*) denote the number of counts recorded on day *d *in a given syndrome. Several SS systems [6-8] have reported models similar to the following:

C(d)~Poisson(∑i=17ciIi(d)+[c8+c9d]+[c10cos(2πd365.25)+c11sin(2πd365.25)])     (1),
 MathType@MTEF@5@5@+=feaafiart1ev1aaatCvAUfKttLearuWrP9MDH5MBPbIqV92AaeXatLxBI9gBaebbnrfifHhDYfgasaacH8akY=wiFfYdH8Gipec8Eeeu0xXdbba9frFj0=OqFfea0dXdd9vqai=hGuQ8kuc9pgc9s8qqaq=dirpe0xb9q8qiLsFr0=vr0=vr0dc8meaabaqaciaacaGaaeqabaqabeGadaaakeaacqWGdbWqcqGGOaakcqWGKbazcqGGPaqkcqGG+bGFcqqGqbaucqqGVbWBcqqGPbqAcqqGZbWCcqqGZbWCcqqGVbWBcqqGUbGBcqGGOaakdaaeWbqaaiabbogaJnaaBaaaleaacqqGPbqAaeqaaOGaemysaK0aaSbaaSqaaiabdMgaPbqabaGccqGGOaakcqWGKbazcqGGPaqkaSqaaiabdMgaPjabg2da9iabigdaXaqaaiabiEda3aqdcqGHris5aGGaaOGae83kaSIaee4waSLaee4yam2aaSbaaSqaaiabbIda4aqabaGccqWFRaWkcqqGJbWydaWgaaWcbaGaeeyoaKdabeaakiabbsgaKjabb2faDjab=TcaRiabbUfaBjabbogaJnaaBaaaleaacqqGXaqmcqqGWaamaeqaaOGaee4yamMaee4Ba8Maee4CamNaeeikaGYaaSaaaeaacqqGYaGmiiGacqGFapaCcqqGKbazaeaacqqGZaWmcqqG2aGncqqG1aqncqqGUaGlcqqGYaGmcqqG1aqnaaGaeeykaKIae83kaSIaee4yam2aaSbaaSqaaiabbgdaXiabbgdaXaqabaGccqqGZbWCcqqGPbqAcqqGUbGBcqqGOaakdaWcaaqaaiabbkdaYiab+b8aWjabbsgaKbqaaiabbodaZiabbAda2iabbwda1iabb6caUiabbkdaYiabbwda1aaacqqGPaqkcqqGDbqxcqqGPaqkcaWLjaGaaCzcamaabmaabaGaeeymaedacaGLOaGaayzkaaGaeiilaWcaaa@8569@

where the notation

a) ∑i=17ciIi(d)
 MathType@MTEF@5@5@+=feaafiart1ev1aaatCvAUfKttLearuWrP9MDH5MBPbIqV92AaeXatLxBI9gBaebbnrfifHhDYfgasaacH8akY=wiFfYdH8Gipec8Eeeu0xXdbba9frFj0=OqFfea0dXdd9vqai=hGuQ8kuc9pgc9s8qqaq=dirpe0xb9q8qiLsFr0=vr0=vr0dc8meaabaqaciaacaGaaeqabaqabeGadaaakeaadaaeWbqaaiabbogaJnaaBaaaleaacqqGPbqAaeqaaOGaemysaK0aaSbaaSqaaiabdMgaPbqabaGccqGGOaakcqWGKbazcqGGPaqkaSqaaiabdMgaPjabg2da9iabigdaXaqaaiabiEda3aqdcqGHris5aaaa@3BC6@ captures the day-of-the-week effect, where, *I*_*i*_(*d*) equals 1 when day *d *is the *i-*th day of the week and equals zero otherwise, and the seven coefficients {*c*_*i*_} are constrained to sum to zero, resulting in six freely varying day-of-week parameters,

b) [c_8 _+ c_9_*d*] captures a long term linear effect,

c) [c10cos(2πd365.25)+c11sin(2πd365.25)]
 MathType@MTEF@5@5@+=feaafiart1ev1aaatCvAUfKttLearuWrP9MDH5MBPbIqV92AaeXatLxBI9gBaebbnrfifHhDYfgasaacH8akY=wiFfYdH8Gipec8Eeeu0xXdbba9frFj0=OqFfea0dXdd9vqai=hGuQ8kuc9pgc9s8qqaq=dirpe0xb9q8qiLsFr0=vr0=vr0dc8meaabaqaciaacaGaaeqabaqabeGadaaakeaacqqGBbWwcqqGJbWydaWgaaWcbaGaeeymaeJaeeimaadabeaakiabbogaJjabb+gaVjabbohaZjabbIcaOmaalaaabaGaeeOmaidcciGae8hWdaNaemizaqgabaGaee4mamJaeeOnayJaeeynauJaeeOla4IaeeOmaiJaeeynaudaaiabbMcaPGGaaiab+TcaRiabbogaJnaaBaaaleaacqqGXaqmcqqGXaqmaeqaaOGaee4CamNaeeyAaKMaeeOBa4MaeeikaGYaaSaaaeaacqqGYaGmcqWFapaCcqWGKbazaeaacqqGZaWmcqqG2aGncqqG1aqncqqGUaGlcqqGYaGmcqqG1aqnaaGaeeykaKIaeeyxa0faaa@5592@ captures a seasonal component, where the average number of days per year is 365.25, with the coefficients c_10 _and c_11_determining the timing and amplitude of a seasonal effect.

**Figure 1:**
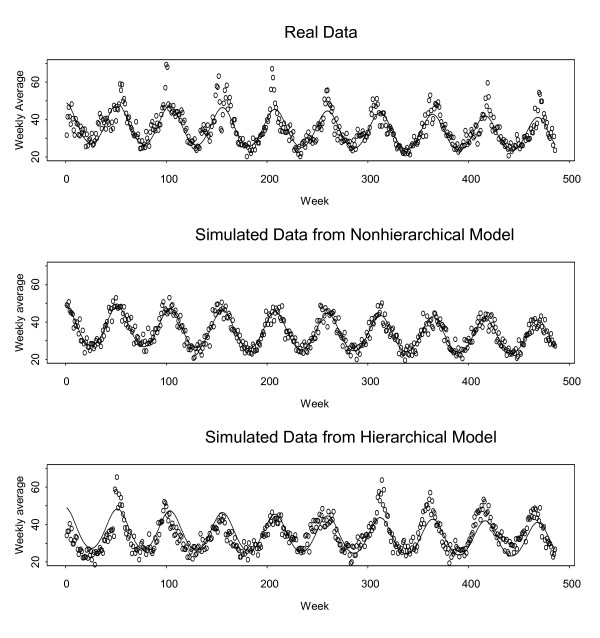
**Average daily respiratory counts, by week **(top) Real data; (middle) Data simulated from Eq. (1) (nonhierarchical model with larger-than-Poisson variance); (bottom) Data simulated from Eq. (2) (hierarchical model with larger-than-Poisson variance). In all 3 plots, the weekly averages begin on Jan 31, 1994 and end on May 31, 2003. The smooth curve is the Eq. (1) fit to all nine years of respiratory data.

Figure 1 (top) is a plot of the average daily count by week from Jan 31, 1994 through May 31, 2003 for the respiratory syndrome from the Albuquerque University Hospital BSafer system [6]. The smooth curve (in all three plots) is the fit to Eq. (1) using all nine years of data.

If the model based on fitting Eq. (1) to the daily counts fit the data well, then its predictions would also fit the average daily count by week very well, but notice that this is not the case. First, a violation of Eq. (1) is that not all years have the same seasonal peak onset, shape, or duration. For example, there are small groups of large positive forecast errors associated with several of the annual seasonal peaks. Typically, this effect is neglected, and the resulting forecast errors from fitting Eq. (1) exhibit serial correlation arising from runs of positive (and negative) forecast errors. Using the log transform improves the fit, but does not eliminate the serial correlation. Second, even in the absence of this violation, the variance-to-mean ratio is typically considerably larger than one, so it is preferable to use a model that is over-dispersed relative to the Poisson (having larger variance-to-mean ratio), such as the Negative Binomial [4].

The middle plot of Figure 1 displays data simulated with a mean given by fitting Eq. (1), but using Negative Binomial variation in order to have approximately the same variance-to-mean ratio as in the real data. One of the consequences of the "one season fits all" assumption described above is that data simulated from the model are likely to lead to optimistic performance claims regarding outbreak DPs. We propose a hierarchical model that largely overcomes this problem. The bottom plot of Figure 1 illustrates data from the hierarchical model (see the next subsection for details of this model). Qualitatively, the bottom plot is more similar to the real data than is the middle plot.

**Figure 2:**
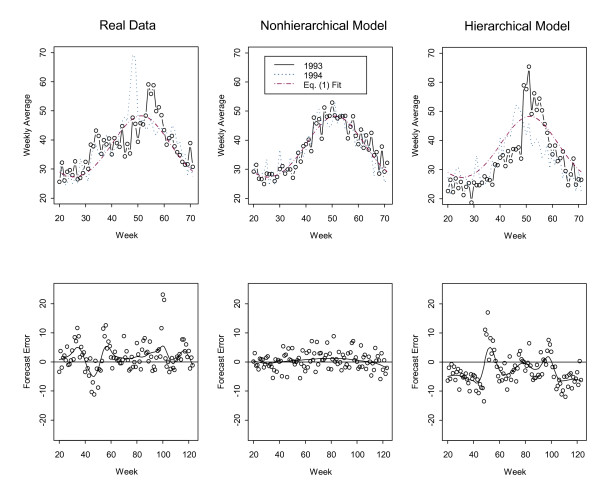
**Average daily average respiratory count, by week, for week 20 through week 71**. The same data as in Figure 1, but only for week 20 through week 71 in 1993 and for week 20 through week 71 in 1994. Note that the peak onset, shape, and duration varies each year in the real data (top left) and in data simulated from the hierarchical model (top right), but not in data simulated from the nonhierarchical model (top middle). The forecast errors arising from using Eq. (1) to forecast (Method 1) and a smooth curve fit to the errors illustrate strong serial correlation in the corresponding bottom left and right plots (and very mild or negligible serial correlation in the middle plot). Each bottom plot shows forecast errors for the same period, weeks 20 through 123, without overlaying successive years.

Figure 2 shows real (top left) average daily counts by week for 1993 and for 1994, beginning at week 20 so that the peak will appear in the middle of the plot, data simulated from the nonhierarchical (top middle) model with Negative Binomial variation to model the larger-than-Poisson variance in the real data, and from the hierarchical (top right) model, also with larger-than-Poisson variance. This again shows that both the real data and the data simulated from the hierarchical model illustrate lack of fit to the "one season fits all" assumption that is implicit in Eq. (1). For clarity, each bottom plot shows forecast errors (arising in all three bottom plots from fitting the non-hierarchical Eq. (1) to the corresponding data) for the same period, weeks 20 through 123, without overlaying successive years. These forecast errors and a smooth curve fit to the errors illustrate serial correlation in the corresponding bottom left and right plots. In contrast, the errors exhibit much less (statistically negligible) serial correlation in the bottom middle plot.

This lack of fit leads to undesirable behavior of sequential tests that monitor for runs of successive positive forecast errors, such as Page's test [9-11]. Page's test is based on *P*(*d*) = max(*P*(*d *- 1) + *e*(*d*) - *k*, 0) and alarms (flags a count or sequence of counts as being unusually high relative to the forecasted value(s)) if *P(d) *exceeds a threshold *h *that is set to achieve the desired false alarm probability. The control parameter *k *is usually chosen to be one half of the mean shift that is important to detect, and the forecast errors *e(d) *are typically scaled to have unit variance.

Page's test is often an effective strategy for detecting multi-day outbreaks [11], but Page's test does not perform well in the presence of this type of serial correlation in the errors. The runs of positive errors (such as can arise when the high-count season starts earlier than its average start date) inflate the value of Page's test and undesirably force the use of a higher alarm threshold *h*, which reduces the DP for true outbreaks.

### Simulated data: hierarchical modeling with over-dispersion

The nonhierarchical model in Eq. (1) is potentially useful for some SS data, and perhaps also for our respiratory counts in particular as a relatively simple, but rough model for routine monitoring. It has the shortcoming, however, of describing each season in a one-size-fits-all fashion, which means that Eq. (1) incorrectly implies that each year's seasonal effect has the same onset, duration, and magnitude. As a consequence, forecast errors arising from using Eq. (1) to forecast real data reflect modeling imperfections (leading for example to serial correlation in the errors) as well as purely random variability. In contrast, forecast errors arising from using Eq. (1) to forecast data simulated from Eq. (1) (the nonhierarchical model) tend to have unrealistically ideal behaviour (Figures 1 and 2) with negligible serial correlation. In order for simulated data to lead to forecast errors that better represent those resulting from real data we introduce a hierarchical model.

Bayesian hierarchical modeling in our context is a generalization of linear modeling in which model parameters such as the seasonal and day-of-week coefficients and other parameters follow a probability distribution whose parameters may be estimated from the data [12]. For the hierarchical model used here, the parameters characterizing the distribution of a given coefficient or a given parameter in Eq. (2) below are not fixed, but instead are assigned a prior distribution (the "hyperprior" in this context) that can be estimated from other independent data. This hyperprior is in the hierarchy of modeling assumptions relating data and parameters [12].

Our hierarchical model is similar to Eq. (1) except the seasonal peak is modeled using a scalable Gaussian function, in contrast with the fixed-width and fixed-location sine and cosine harmonics [13]. Also, the underlying baseline count rate changes linearly within a year, as opposed to behaving linearly over a longer time period. In particular, the expected number of counts on day *d *in year *y *is modeled as

E(Cd)=by(d)+ayσyφ(d−Δyσy)+∑i=17ciIi(d)     (2),
 MathType@MTEF@5@5@+=feaafiart1ev1aaatCvAUfKttLearuWrP9MDH5MBPbIqV92AaeXatLxBI9gBaebbnrfifHhDYfgasaacH8akY=wiFfYdH8Gipec8Eeeu0xXdbba9frFj0=OqFfea0dXdd9vqai=hGuQ8kuc9pgc9s8qqaq=dirpe0xb9q8qiLsFr0=vr0=vr0dc8meaabaqaciaacaGaaeqabaqabeGadaaakeaacqWGfbqrcqGGOaakcqWGdbWqdaWgaaWcbaGaemizaqgabeaakiabcMcaPiabg2da9iabdkgaInaaBaaaleaacqWG5bqEaeqaaOGaeiikaGIaemizaqMaeiykaKIaey4kaSYaaSaaaeaacqWGHbqydaWgaaWcbaGaemyEaKhabeaaaOqaaGGaciab=n8aZnaaBaaaleaacqWG5bqEaeqaaaaakiab=z8aMjabcIcaOmaalaaabaGaemizaqMaeyOeI0IaeuiLdq0aaSbaaSqaaiabdMha5bqabaaakeaacqWFdpWCdaWgaaWcbaGaemyEaKhabeaaaaGccqGGPaqkcqGHRaWkdaaeWbqaaiabbogaJnaaBaaaleaacqqGPbqAaeqaaOGaemysaK0aaSbaaSqaaiabdMgaPbqabaGccqGGOaakcqWGKbazcqGGPaqkaSqaaiabdMgaPjabg2da9iabigdaXaqaaiabiEda3aqdcqGHris5aOGaaCzcaiaaxMaadaqadaqaaiabikdaYaGaayjkaiaawMcaaiabcYcaSaaa@6156@

where

a) by(d)=by−1+d365(by−by−1)
 MathType@MTEF@5@5@+=feaafiart1ev1aaatCvAUfKttLearuWrP9MDH5MBPbIqV92AaeXatLxBI9gBaebbnrfifHhDYfgasaacH8akY=wiFfYdH8Gipec8Eeeu0xXdbba9frFj0=OqFfea0dXdd9vqai=hGuQ8kuc9pgc9s8qqaq=dirpe0xb9q8qiLsFr0=vr0=vr0dc8meaabaqaciaacaGaaeqabaqabeGadaaakeaacqWGIbGydaWgaaWcbaGaemyEaKhabeaakiabcIcaOiabdsgaKjabcMcaPiabg2da9iabdkgaInaaBaaaleaacqWG5bqEcqGHsislcqaIXaqmaeqaaOGaey4kaSYaaSaaaeaacqWGKbazaeaacqaIZaWmcqaI2aGncqaI1aqnaaGaeiikaGIaemOyai2aaSbaaSqaaiabdMha5bqabaGccqGHsislcqWGIbGydaWgaaWcbaGaemyEaKNaeyOeI0IaeGymaedabeaakiabcMcaPaaa@482F@ models the changing baseline count rate by linearly interpolating between the current (*b*_*y*_) and the previous (*b*_*y*-1_) off-peak baseline,

b) *a*_*y *_is the scaled peak amplitude for year *y*,

c) *φ *is the probability density for a standard normal random variable,

φ(z)=12πe−z2/2,
 MathType@MTEF@5@5@+=feaafiart1ev1aaatCvAUfKttLearuWrP9MDH5MBPbIqV92AaeXatLxBI9gBaebbnrfifHhDYfgasaacH8akY=wiFfYdH8Gipec8Eeeu0xXdbba9frFj0=OqFfea0dXdd9vqai=hGuQ8kuc9pgc9s8qqaq=dirpe0xb9q8qiLsFr0=vr0=vr0dc8meaabaqaciaacaGaaeqabaqabeGadaaakeaaiiGacqWFgpGzcqGGOaakcqWG6bGEcqGGPaqkcqGH9aqpdaWcaaqaaiabigdaXaqaamaakaaabaGaeGOmaiJae8hWdahaleqaaaaakiabdwgaLnaaCaaaleqabaGaeyOeI0IaemOEaO3aaWbaaWqabeaacqaIYaGmaaWccqGGVaWlcqaIYaGmaaGccqGGSaalaaa@3E47@

d) Δ_*y *_denotes the time of the peak for year *y*,

e) *σ*_*y *_corresponds to the duration of the peak in year *y*, and

f) the day-of-week effects are included using the indicator function ∑i=17ciIi(d)
 MathType@MTEF@5@5@+=feaafiart1ev1aaatCvAUfKttLearuWrP9MDH5MBPbIqV92AaeXatLxBI9gBaebbnrfifHhDYfgasaacH8akY=wiFfYdH8Gipec8Eeeu0xXdbba9frFj0=OqFfea0dXdd9vqai=hGuQ8kuc9pgc9s8qqaq=dirpe0xb9q8qiLsFr0=vr0=vr0dc8meaabaqaciaacaGaaeqabaqabeGadaaakeaadaaeWbqaaiabbogaJnaaBaaaleaacqqGPbqAaeqaaOGaemysaK0aaSbaaSqaaiabdMgaPbqabaGccqGGOaakcqWGKbazcqGGPaqkaSqaaiabdMgaPjabg2da9iabigdaXaqaaiabiEda3aqdcqGHris5aaaa@3BC6@ as in Eq. (1). Other effects such as holiday effects can easily be included in either Eq. (1) or Eq. (2). In addition, the variance-to-mean ratio is not constrained to be one. Instead, we let variance(Cd|E(Cd))=1+ψψE(Cd)
 MathType@MTEF@5@5@+=feaafiart1ev1aaatCvAUfKttLearuWrP9MDH5MBPbIqV92AaeXatLxBI9gBaebbnrfifHhDYfgasaacH8akY=wiFfYdH8Gipec8Eeeu0xXdbba9frFj0=OqFfea0dXdd9vqai=hGuQ8kuc9pgc9s8qqaq=dirpe0xb9q8qiLsFr0=vr0=vr0dc8meaabaqaciaacaGaaeqabaqabeGadaaakeaacqqGOaakcqWGdbWqdaWgaaWcbaGaemizaqgabeaakiabcYha8jabdweafjabcIcaOiabdoeadnaaBaaaleaacqWGKbazaeqaaOGaeiykaKIaeiykaKIaeyypa0ZaaSaaaeaacqaIXaqmcqGHRaWkiiGacqWFipqEaeaacqWFipqEaaGaemyrauKaeiikaGIaem4qam0aaSbaaSqaaiabdsgaKbqabaGccqGGPaqkaaa@43AF@, where as *ψ *→ ∞, the variance-to-mean ratio approaches 1. Graves and Picard [13] provide additional mathematical details including hyperprior specifications.

In our context, there is non-negligible year-to-year variation in each seasonal peak (Figs 1 and 2) and baseline, and also in the day-of-week effects. On the basis of the hierarchical model, we generate counts for a particular Tuesday, say, as follows. First, we randomly choose an off-peak baseline *b*_*y *_for the given year (and for the year prior so that linear interpolation of two baseline off-peak means can be applied), then randomly choose parameters *a*_*y*_, *σ*_*y*_, and Δ_*y *_so that the term ayσyφ(d−Δyσy)
 MathType@MTEF@5@5@+=feaafiart1ev1aaatCvAUfKttLearuWrP9MDH5MBPbIqV92AaeXatLxBI9gBaebbnrfifHhDYfgasaacH8akY=wiFfYdH8Gipec8Eeeu0xXdbba9frFj0=OqFfea0dXdd9vqai=hGuQ8kuc9pgc9s8qqaq=dirpe0xb9q8qiLsFr0=vr0=vr0dc8meaabaqaciaacaGaaeqabaqabeGadaaakeaadaWcaaqaaiabdggaHnaaBaaaleaacqWG5bqEaeqaaaGcbaacciGae83Wdm3aaSbaaSqaaiabdMha5bqabaaaaOGae8NXdyMaeiikaGYaaSaaaeaacqWGKbazcqGHsislcqqHuoardaWgaaWcbaGaemyEaKhabeaaaOqaaiab=n8aZnaaBaaaleaacqWG5bqEaeqaaaaakiabcMcaPaaa@3F6D@, which describes the seasonal increase, if any, can be computed for the given time of year, then add the Tuesday effect, *c*_*i *_with the value of *i *denoting Tuesday, and finally, simulate a count from a distribution that is over-dispersed relative to the Poisson to an extent determined by *ψ*, but has an expected value corresponding to the randomly-chosen effects. This approach assumes, as we empirically verified, that the coefficients are approximately mutually independent.

Clearly, this hierarchical, synthetic data generation scheme differs from that given by Eq. (1), which assumes each year's season is the same (with respect to onset, duration, and magnitude of the peak, except for the drifting baseline). Hierarchical methods eliminate the "one season fits all" modeling assumption by allowing, for example, each year to have its own linear baseline, its own onset of peak activity, its own seasonal duration, and its own peak magnitude.

Fitting the hierarchical model to the BSafer [6] respiratory counts illustrates the year-specific nature of the seasonal pattern. On average, the respiratory counts peak on January 22, with a season-to-season standard deviation of 12 days. The durations of individual seasons, defined in terms of the standard deviations for the Gaussian-shaped peaks, vary by a factor of two over the monitoring period. And there is no apparent relation between the time that the peak occurs and the magnitude of the season. Such details provide insight into the data that are obscured by "one season fits all" models.

YADAS was used to estimate the hierarchical model parameters using Markov Chain Monte Carlo (MCMC) [14]. The MCMC strategy is to simulate observations from the joint posterior distribution for all model coefficients and parameters (*c*_1_, ..., *c*_7_, and *ψ*, and *b*_*y*_, *a*_*y*_, *σ*_*y*_, Δ_*y*_, for each year). We did this using YADAS by randomly choosing starting coefficient values from the parameters' prior distributions, proposing small changes to each coefficient, and accepting the changed values with a probability determined by the ratio of the posterior probability of the data given these candidate coefficient values to the posterior probability of the data given the current coefficient values. After sufficiently many iterations, this Markov Chain produces a sequence of observations from the posterior distribution for the parameters, which can be summarized, for example, by calculating the mean and standard deviation of the generated sequence of each parameter's values [12]. Given the joint posterior probability distribution for the coefficients, we generate data for a given Tuesday in a given year, for example, as described above.

Hierarchical models for real time SS cshould be considered, but at a computational cost. To capture the peak time and magnitude of an ongoing season, the model must be updated frequently (e.g., weekly), involving lengthy runs of specialized software, but perhaps such a method could improve upon simpler methods for detecting anomalous outbreaks. Currently, nonhierarchical models such as Eq. (1) are among the typical approaches for real time SS [6-8]. We introduce the hierarchical model here because, compared to fitting to Eq. (1), it is a better model-based summary of the real data, and data simulated from the hierarchical model provides as realistic or more realistic estimates of outbreak DPs as detailed in the next section.

### Study description

Hutwagner et al. [4,5] reported results from a comparison-of-forecasting-methods study applied to data simulated from a model similar to Eq. (1), using Negative Binomial variation to capture over-dispersion relative to Poisson dispersion. One purpose for our work is to suggest that conclusions from studies that rely exclusively on simulated one-season-fits-all data may be vulnerable to optimistic claims arising from unmodeled data features, such as violation of the "one-season-fits-all" assumption. As we have discussed, our outbreak-free training data contains a yearly seasonal effect that is not considered to be an outbreak that should be detected. In other contexts a goal might be to determine whether SS data could more rapidly detect the onset of the influenza season than existing surveillance methods based on definitive influenza test results [6].

Using real respiratory syndrome counts collected from the BSafer system [6] from 2/1/94–5/31/03, we varied the length of training data from one to eight years, applied a sequential test to the forecast errors arising from each of eight forecasting methods, and evaluated the DP on the basis of injected synthetic outbreaks and the use of Page's test to detect anomalous values. We did the same for each of the two corresponding simulated data sets. The nonhierarchical model's simulated data set assumed that "one season fits all." The hierarchical model's simulated data set did not assume that "one season fits all," as described above. The following paragraphs provide more detail.

The forecasting methods in [4,5] included moving averages and historical averages using periods that bracket the period being forecasted. This study includes the same forecasting methods, plus adds a method that fits the training data to Eq. (1), and adds an exponentially weighted moving average (EWMA) method [15]. We include results for the real data and for data simulated from one realization each of the hierarchical and nonhierarchical models.

Because the forecast errors from each method were analyzed using Page's test (see the Methods section), the methods differ only on the basis of the forecasting method. Also, because the log transform improves the fit to Eq. (1), we computed forecast errors *e*(*d*) from a fit to *S*(*d*) = log(*C*(*d*) + 1) rather than to *C*(*d*) for all forecast methods. These forecast errors were scaled to have unit variance prior to application of Page's test.

The eight forecast methods are as follows. Method 1 uses the fit to Eq. (1) as the forecast. Method 2 is EWMA, where the day *d *forecast for the series *S *is *F(d) *= *α*S(*d*-1) + (1 - *α*)*F(d - 1)*. The coefficient *α *was chosen to minimize the forecast error variance in the training data, and was approximately 0.2 in all training cases. Method 3 is a 7-day moving average, using a gap of 1 day (so the forecast for day 9 is the average of days 1 to 7, etc.). Method 4 is a 7-day moving average using a gap of 3 days. Method 5 uses the historical average (in the training data) of days *d*-1, *d*, and *d *+ 1 to predict day *d*. Method 6 uses the historical average of three 4-day windows: the 4-day window containing day *d *and the 4-day windows before and after this 4-day window containing day *d*. Method 7 is the same as Method 5, except uses only day *d *in the training data to predict day *d *in the test data. Method 8 is the same as Method 6, except uses only the historical average of a 4-day window that contains day *d*. Methods 3–8 or minor variations thereof were evaluated in [4].

The data for our study exhibits a strong day-of-week effect. Monday counts are higher than Friday counts by an average of approximately 7, but with substantial variation. For example, in nearly 25% of the weeks, Monday counts are lower than Friday counts. To accommodate day-of-week effects, the moving average results reported in [4] used averages of 7-day windows. We experimented with 1 to 10 day windows and various gaps (see below) and empirically confirmed that with BSafer [6] data, a 7-day window is the most effective size. Using a 7-day window ensures that each day of the week contributes to the average.

The training data sets include: (1) the real data [6] shown in Figure 1, (2) simulated data from the non-hierarchical model fit to the real data having Negative Binomial variation around expected counts arising from Eq. (1), and (3) simulated data that follows the hierarchical model fit to the real data allowing yearly variation in the seasonal coefficients as described. For both the hierarchical and non-hierarchical sets of simulated data, parameters were chosen in order to approximately match the variance-to-mean ratio of 1.8 observed in the real data.

For each of the 3 data sets, for each case, as defined by the number of training years, 1000 outbreaks were simulated, with each outbreak having random (uniform) duration of 1 to 20 days, beginning at a random (uniform) day (from 1 to the number of days (365 or 366) in the test year), and shaped like a lognormal distribution with a rapid rise and slow decline in counts. Each simulated outbreak was inserted into the test year simply by adding the simulated outbreak to the test data beginning at the random starting day. Outbreaks lasting multiple days that began near day 365 were truncated to fit within the testing year. The total number of injected counts over the duration of each outbreak also varied randomly (uniformly) from 5 to 50 times the standard deviation of the forecast errors arising from using forecast method 1 (see below) in the training data for all eight years. This outbreak size was chosen to give reasonably large DP for a false alarm rate (a false alarm is any alarm occurring in the training data, which contains no outbreaks) of one per year. Because most outbreaks were multiple days, this study reports results only for a sequential test (Page's) applied to the forecast errors. Other tests that scan for multiple-day outbreaks could be considered [16].

Training data consisted of various training cases, defined on the basis of whether the first year, the first two years, the first three years, etc. were used for training. In each training case, the test year for DP assessment and for comparing the nominal (anticipated false alarm rate on the basis of the false alarm rate on the training data) to the actual false alarm rate was the year immediately following the end of the training period. The year for selecting Page's threshold *h *to achieve one false alarm per year was the final year of the training data. For example, in the case with two years of training data, year two is used to choose the threshold *h *for Page's test, and year three is used to compare the actual to the nominal false alarm rate (prior to outbreak insertion) and for assessing DPs (following outbreak insertion).

## Results

### Study results

Table 1 lists 100 times the DP by method, length of training data, and data set. Traditionally, Methods 5–8 have been used with at least 3 years of training data [4]; therefore the average DPs summarized below and in Table 1 refer to averages over the cases having 3–8 years of training data for Methods 5–8 and cases having 1–8 years of training data for Methods 1–4.

**Table 1:** Detection probabilities (multiplied by 100) by forecast method, length of training data, and data set.

	Forecast Method							
	
Years of Training Data	1 Eq. (1)	2 EWMA	3 Avg1	4 Avg2	5 HistAvg1	6 HistAvg2	7 HistAvg3	8 HistAvg4
1 year	88,86,100	93,92,88	92,72,86	89,78,84	81,74,84	84,75,100	71,61,92	79,74,100
2 years	69,79,49	94,91,90	92,73,89	90,78,89	74,61,62	77,65,68	70,53,55	73,63,64
3 years	70,99,95	89,93,93	87,92,91	81,88,89	66,90,65	69,94,82	61,89,69	68,90,66
4 years	83,94,99	89,90,91	89,89,89	81,89,71	79,89,97	78,90,97	76,88,95	66,92,94
5 years	92,90,68	91,95,85	88,92,72	84,91,75	76,83,73	77,86,75	73,80,70	76,83,75
6 years	75,91,96	90,95,86	90,88,82	86,72,66	68,84,93	68,90,93	66,84,93	68,89,90
7 years	96,92,66	93,86,91	92,80,92	90,88,87	97,90,72	97,92,74	96,90,70	96,91,73
8 years	82,93,96	95,96,91	94,91,84	92,89,81	87,90,94	88,92,94	88,88,94	89,87,93
Average	83,**91**,84	92,92,89	91,85,85	87,84,81	79,**88**,82	79,**91**,86	77,**86**,82	77,**89**,82

The (*x*,*y*,*z*) entry in each cell denotes 100 times the DP (with a false alarm rate of one per year) for the real data, data simulated from the nonhierarchical model, and data simulated from the hierarchical model, respectively. Individual entries are each based on 1000 simulated outbreaks, so their confidence limits are approximately ± 2. In the last row, boldface entries indicate optimistic DPs attributable to using data simulated from the nonhierarchical model. The eight methods are based on applying Page's test to forecast errors based on forecasts from: (1) a fit to Eq. (1), (2) EWMA, (3) moving average with a 1-day gap, (4) moving average with a 3-day gap, (5) historical average using day *d*, *d*-1, *d*+1 in the training data to predict day *d *in the testing data, (6) historical average using three 4-day windows, (7) historical average using day *d*, and the (8) historical average using one 4-day window.

Individual entries in Table 1 are each based on 1000 simulations, so their confidence limits are approximately ± 2 (on the basis of the normal approximation to the binomial). Upon repeating the set of 1000 simulations and making each comparison twice, we found that when comparisons among the eight methods or three data sets are made using averages, the values of the paired differences are repeatable to within approximately ± 1.

### Study summary

Table 1 can be summarized as follows. The DPs tend to be highest for data set 2 (nonhierarchical model), as expected. The average DP is 0.83 for data set 1 (real data), 0.88 for data set 2 (nonhierarchical model), and 0.84 for data set 3 (hierarchical model). The DPs from data set 3 are generally closer to those from the real data (average DP difference of 0.01) than are the DPs from the nonhierarchical model (average DP difference of 0.05). The average DPs by method are given in the last row, and indicate that Methods 1 and 5–8 have large positive differences (optimistic bias) between the DP for data set 2 and the DP for data set 1. The moving average methods (Methods 2–4) show negligible evidence of optimism and Methods 3 and 4 actually show evidence of pessimistic bias. There are occasional bad test years (rows 3 and 6) that have low DP even if there are multiple training years. The boldface entries in the final row indicate instances in which DP estimates based on data simulated from the non-hierarchical model are optimistic compared to the real data and data simulated from the hierarchical data.

## Discussion

Although sufficient real data was available, [4] and [5] used simulated data to estimate DPs. Whether DPs on simulated data will be close to those on real data is always an important question, and significant differences in DPs between simulated and real data as found here can lead to improvements in the simulation model.

We do not suggest that forecast Methods 1 through 8 should in general be ranked according to their DPs reported here. This study is based on one data set with one type of simulated outbreak (approximately lognormal with varying numbers of days), one type of sequential test (Page's) and one nominal false alarm rate (one false alarm per year). In fact, Brillman et al. [6] illustrated relatively good performance of Method 1 when a higher false alarm rate is allowed and smaller outbreaks were injected. We do want to emphasize again, however, that Method 1 is among the most prone of the methods considered here to optimistic claims arising from using a nonhierarchical model. On the other hand, if seasonal and/or day-of-week effects were more consistent, then Method 1 should perform the best on the real data (and on data simulated from the either the corresponding hierarchical or nonhierarchical models) because Eq. (1) would be a better description of the real data.

The moving average forecast methods (Methods 2–4) performed relatively well on all data sets, showing relatively high DP and less optimistic bias (no boldface entries in the last row of Table 1) when comparing DPs from the real data or from the hierarchical model to DPs from data simulated from the non-hierarchical model. Among Methods 2–4, the EWMA method is the best performer on the real data, although there is no statistical difference among Methods 2–4 for some cell entries in Table 1.

Recall that we selected alarm thresholds that corresponded to one false alarm per year in the year prior to the test year for which we reported the DP. This raises the issue that alarm thresholds selected on the basis of limited training and/or testing data might not have the desired (nominal) false alarm rate in any given test year. All forecasting methods are vulnerable to having the actual false alarm rates substantially different from the nominal alarm rates. For example, the average (over all training cases and methods) false alarm rates in the test year when thresholds were selected on the basis of the previous test year were 1.3, 1.1, and 3.1, respectively for data sets 1, 2, and 3, with Methods 1 to 8 having an average over all training cases and data sets of 4.0, 0.9, 0.8, 0.8, 2.1, 2.5, 2.1, and 1.9 false alarms, respectively. Therefore, although these are reasonably close to the nominal rate of 1 per year, this effect is potentially of practical importance. Although we focus on the respective DPs, this provides another suggestion that Methods 1 and 5–8 do not perform well relative to Methods 2–4, unless a "one season fits all" model adequately describes the data.

## Conclusion

For the data we analyze: (1) the "one season fits all " assumption is violated, and DP performance claims based on simulated data that assume "one season fits all," for the forecast methods considered, except for moving average methods, tend to be optimistic, and (2) moving average methods based on relatively short amounts of training data are competitive on all three data sets, but are particularly competitive on the real data and on data from the hierarchical model, which are the two data sets that violate the "one season fits all" assumption.

More specifically, the DPs for all methods except for moving average methods on the real data are generally lower than those for data simulated from the nonhierarchical model, and closer to those for data simulated from the hierarchical model. This suggests that when estimating DPs, there are violations of the "one season fits all" assumption that are important to model in the respiratory counts from BSafer [6]. Moving average methods for this data have high DPs, negligible observed tendency toward optimistic DP claims, and achieved an actual false alarm rate of slightly less than the nominal rate of one per year.

In some situations, only a few years of training data are available. This is often a reason for using simulated data for estimating DPs. This was also one reason for considering moving average predictions in [4]. In addition, moving average methods are known to be somewhat robust to various modeling assumption violations [13], and to be competitive, especially when training data is very limited, or when there are relatively frequent changes in various aspects of the data, such as in the off-peak baseline count rate. On the other hand, moving average methods suffer when forecast errors from later stages of multi-day outbreaks are reduced once the early-stages of the outbreak increase the moving average forecast, and if there are strong day-of-week effects that are not accommodated.

One approach is to use several forecasting methods on each particular data set. For example, BioSense [8] currently includes both moving average methods and a method involving fitting a model similar to Eq. (1). However, the false alarm rate necessarily increases when multiple forecasting methods are used simultaneously.

We have not yet considered whether a hierarchical-model-based forecasting method could be developed. It is possible that such a method could improve upon simpler methods for detecting anomalous outbreaks such as those considered here. For example, suppose elevated count rates due to innocent seasonal effects occur somewhat earlier in a given training year than predicted on the basis of the Bayesian posterior distribution of peak season start times. Such elevated counts could lead to a sequence of large positive forecast errors if monitoring were based on the non-hierarchical model, but only to moderately large positive forecast errors if based on the hierarchical model. Using such training data, the required decision thresholds for any sequential test (such as Page's test) would tend to be more elevated for the nonhierarchical model than for the hierarchical model. This would lead to lower DPs for the nonhierarchical model when true outbreaks occur.

Overall, for the data studied here, nonhierarchical models tended to yield optimistic performance assessments compared to assessments based on real data. Hierarchical models aid our understanding of the data, and provide more realistic model-based alarm thresholds and DP assessments. Moving average methods tend to be more robust than model-based methods when those model-based methods are based on simplistic (i.e., nonhierarchical) assumptions.

## Competing interests

The author(s) declare that they have no competing interests.

## Authors' contributions

SM and TB implemented the methods and performed the simulations. TG and RP developed the hierarchical model; TG estimated its parameters for the BSafer data.

NH and RK provided technical assistance and all authors helped write the manuscript.

All authors read and approved the final manuscript

## Pre-publication history

The pre-publication history for this paper can be accessed here:


